# A survey of hospice day services in the United Kingdom & Republic of Ireland : how did hospices offer social support to palliative care patients, pre-pandemic?

**DOI:** 10.1186/s12904-022-01061-9

**Published:** 2022-10-05

**Authors:** NM Bradley, CF Dowrick, M Lloyd-Williams

**Affiliations:** 1 Research Fellow in Realist Evaluation, Centre for Health & Clinical Research, University of the West of England, Glenside Campus, BS16 1DD., Bristol, United Kingdom; 2 Emeritus Professor, Department of Primary Care and Mental Health, University of Liverpool, Waterhouse Building, L69 3BX., Liverpool, United Kingdom; 3 Professor & Honorary Consultant in Palliative Medicine, Department of Primary Care and Mental Health, University of Liverpool, Waterhouse Building, L69 3BX, Liverpool, United Kingdom

**Keywords:** hospice day services, palliative care, social support

## Abstract

**Introduction:**

Social support is described by patients and other stakeholders to be a valuable component of palliative day care. Less is known about the range of hospice services that have been used in practice that facilitate social support. An online survey aimed to gain an overview of all hospice day services that facilitated social support for adults outside of their own homes.

**Methods:**

An online survey was distributed via email to people involved in managing hospice day services. Questions were asked on hospice characteristics, including staff and volunteer roles. Respondents were asked to identify services they felt offered social support to patients. Data collection took place between August 2017 and May 2018.

**Results:**

Responses were received from 103 hospices in the UK and ROI (response rate 49.5%). Results provide an overview of hospice day and outpatient services that offer social support to patients. These are: multi-component interventions, activity groups, formal support groups, befriending, and informal social activities. Multi-component interventions, such as palliative day care, were the most commonly reported. Their stated aims tend to focus on clinical aspects, but many survey respondents considered these multicomponent interventions to be the ‘most social’ service at their hospice. The survey also identified a huge variety of activity groups, as well as formal therapeutic support groups. Informal ‘social-only’ activities were present, but less common. Over a third of all the services were described as ‘drop in’. Most responding hospices did not routinely use patient reported outcome measures in their ‘most social’ services.

**Conclusions:**

The survey documents hospice activity in facilitating social support to be diverse and evolving. At the time of data collection, many hospices offered multiple different services by which a patient might obtain social support outside of their own home and in the presence of other patients.

**Supplementary Information:**

The online version contains supplementary material available at 10.1186/s12904-022-01061-9.

## Introduction

‘Social support’ is defined as the resource we gain through interaction with others, including tangible, emotional, or informational support, and companionship.[1] We are a social species – social support is an essential need that is tied to our survival.[2] The presence of social support, the absence of positive social relationships, and the subjective feeling of loneliness have each been demonstrated to be predictive of morbidity and mortality.[3–6] Furthermore, this risk exists on a continuum so that the socially isolated are most affected, but those who experience mild to moderate isolation are also affected.[7].

People living with life-limiting illness experience declining social support because their ability to participate in usual activities can be obstructed by changes in mobility, pain and other symptoms, and treatment burden.[8–12] For some, the experience of illness is characterised by increasing social isolation over time.[13] Loneliness in palliative care could stem from threats to personal autonomy, and fears of being or becoming a burden that constrain communication.[11] Personal inauthenticity, interpersonal avoidance, negative healthcare interactions, and the societal stigma of illness and dying contribute multiple layers of patient loneliness.[14–15] Those approaching the end of life with loneliness are more likely to experience depression, feelings of abandonment, and unbearable suffering.[16–19].

The objective of palliative care is to prevent and relieve the suffering of people with life-limiting illness, and their families, by responding simultaneously to physical, psychological, spiritual, social, cultural, and situational needs. Responding to unmet needs for social support could prevent suffering by helping to preserve a sense of purpose and allow threats to be redefined with new meaning and hope.[20] Implications of this for patient care are to maintain social networks where possible, and to arrange relationships with other patients so as to facilitate confidential connections that might lessen the pain of loneliness. One-to-one peer support in cancer care has been reported to be beneficial for some patients [21–23], but there is limited evidence to guide intervention in palliative care [24].

### Hospice day services

Hospices are prominent providers of palliative care; supporting more than 225,000 people in the UK each year to cope with the challenges of illness, dying and bereavement.[25] Most hospices in the UK and ROI are independent charities working within and alongside the local health and social care system. Each hospice might offer numerous services, with the intention to provide holistic and person-centred responses to the varied and fluctuating needs of different patients.[26] Only a minority of hospice services involve staying overnight, with the majority (83%) in 2015 occurring as homecare, outpatient services and hospice day care.[27].

The first hospice day care centre was opened by St Luke’s hospice in Sheffield in 1975– described as a ‘rallying point’ for patients and families to come together to cope with loss.[28] By the turn of the century there were over 220 day hospices in the UK.[29] Some hospice day centres run formal interventions, numerous activity groups and an active social programme, offering informal social interaction during group activities, shared meals, and unstructured social time.[30] Volunteers are commonly involved, contributing socially as well as to creative activities, complementary therapies, transport, counselling and pastoral faith-based support.[31]] Site-level variations in practice are well-established,[32] and continuing to flourish.[33].

Hospice day care is reported to provide a supportive social environment that facilitates engagement in meaningful activities and reduce isolation and dependency.[34] Hospice day services have social objectives such as increased social interaction, decreased isolation, personal growth, a sense of control over the illness experience, and reassurance about the future.[35] It may be that patients experience emotional loneliness at home, which is relieved by spending time with people they perceive to be in similar situations.[36] For socially isolated or homebound patients, getting out for the day and into a new physical environment can be an achievement in itself.[37] But the impact goes beyond just a day out: social interaction, a stimulating environment, and new friendships are confidence-building opportunities for personal growth.[38] Benefits might also extend to the family through informational and emotional support, as well as respite opportunities.[39].

Qualitative work exploring patient experience has led a number of authors to conclude that social interaction is the core of palliative day care.[40–43].However, those with ‘better’ quality of life or positive service experiences may be more likely to be recruited, and participants might feel constrained to give criticism in case it negatively impacts the hospice in some way. Description of the intervention itself is often sparse,[44] and with participants poorly representative of the overall palliative care population.[45] Less is known about people with negative experiences of hospice care, those with poorer quality of life, and those who were not referred, or withdrew from the intervention.[42, 46] This is problematic, because there are unknown mechanisms by which age, gender, and other sociodemographic characteristics influence patient experience and the effectiveness of palliative care interventions.[47].

A systematic review of reported patient and stakeholder perceptions underlined the varied social benefits of attending palliative day care: a sense of belonging and companionship, opportunities to communicate with people in a similar situation, perhaps drawing strength from observing them cope.[48] Despite limitations in the evidence base, it does seem clear that access to the social support within palliative day care is highly valued by the attending patients and by the referring health professionals – although there can be reluctance among clinicians to refer for purely social reasons.[49].

A regional survey of palliative day care reported that most providers included both medical care and social support in their services. When centres were invited to describe whether the model was mainly social or mainly medical, their responses were not found to be associated with reported levels of staffing, management, funding, or activities. Service characteristics did not distinguish between medical and social models in palliative day care, and evidence for a dichotomy between medical and social services was not found.[26] It has been argued that social outcomes should be included in the evaluation of these settings - their objectives and activities go far beyond the health-related and the most prominent aspect reported by attending patients is the opportunity to meet other people.[50] Yet, a recent Delphi study of quality indicators for palliative day services did not include any social structures, processes, or outcomes.[51] It is therefore unclear which hospice day services consider social support to be within their remit.

Other group interventions, social settings, and community-level initiatives are being developed that represent further diversification of hospice day service models. These innovations indicate social support being embedded with other hospice goals - for example, a survey of UK hospices found that public health projects were a priority for most respondents.[52] Initiatives characterised by community members and patients coming together to address end-of-life issues could have social impact at the interpersonal level as well as for the wider population, but there is little reported on the details of these initiatives – especially the extent to which patients themselves are participating and might derive psychosocial benefit from the intervention.

In rehabilitative palliative care, exercise and education groups aim to empower the patient to live actively by achieving functional improvements in time-limited interventions. The incorporation of functional rehabilitation groups into hospice programmes encourages individual independence and personal goals – though groupwork remains a feature. Exercising alongside other patients could be important for the experience and outcomes of the intervention: patients build comradeship and a sense of achievement together and this improves each individual’s self-esteem and confidence beyond the confines of the programme[53]. Shared experiences and verbal encouragement between group members develops a sense of normality and peer support.[54] The social value of being able to get out of the house and participate in (in this case) an exercise class is remarkable, even when the patients attend the hospice for reasons other than social support[53, 54]. Those attending a day hospice rehabilitation programme reported that the intervention was valuable in terms of symptom management, but they would have liked more emotional support and interaction with other patients.[55] This raises uncertainty on the extent to which rehabilitation interventions set out to foster support between attendees.

Day and outpatient services provided by hospices can have a range of components, with different intended outcomes, and variations between sites and between patients. Social support appears significant to patients, but measurement is rare.[44] Less is known about the full range of service models used within hospice day services to facilitate social support, which could differ markedly in their aims, processes, outcomes, and costs. Innovation in service design is ongoing, and some hospice day centres offer a broad menu of options for group support and activity. However, there is sparse evidence available to guide decision-making and much of the informal support offered by hospices has not been quantified.[60].

Hospice care is explicitly holistic, responding to psychosocial concerns as well as symptomatic and spiritual aspects. Clinical or nursing staff and symptom-focused treatment are commonly provided, alongside some form of group involvement or social interaction with other people (including but not necessarily limited to other patients), and thus the opportunity to gain social support. Yet the richness of this area in practice and the significance of social support to patients is not consistently acknowledged. Resolving ambiguity around this field of practice is necessary to inform future research and service design.

### Survey aim

Better intelligence is needed to understand how hospices might offer social support to people with life-limiting illness. Previous surveys have been conducted, but a large and comprehensive overview of ‘social’ services had not yet been established. The survey aimed to gain a broad overview and specific information about hospice day and outpatient services that facilitate social support for people with life-limiting illness; to inform subsequent stages of a mixed-methods project.

Since then, the Covid-19 pandemic has created a stark rupture in hospice service provision and in the lives of many people with long-term health conditions. In recovering from the initial shock of the pandemic, many hospices are now continuing or embarking on service redesign. Much innovation has already taken place, but documentation of practice in the ‘pre-pandemic’ phase of the twenty-first century remains sparse. This online survey asked which services offered social support to adults with life-limiting illness, outside of their own homes. Data collection took place August 2017-May 2018.

## Methods

### Survey design

Surveys can be helpful in illuminating the bigger picture – this enables knowledge exchange and help to focus research endeavours by establishing knowledge of practice.[8] An online survey was considered a practical approach to data collection from a broad geographical area that would enable respondents to complete the survey at a convenient time.

We asked hospices in the UK & ROI how they facilitate social support for adults with life-limiting illness, defined as the opportunity to meet other people outside of their own homes. Respondents were staff responsible for managing or running day services at hospices in the UK & ROI. The research was funded by the Economic & Social Research Council and received ethical approval from the University of Liverpool ethics committee.

SurveyMonkey was used to develop the survey and collect the data. The survey was designed initially by NB and redrafted with MLW and CFD; it was then piloted between June and August 2017 via existing contacts. The intention of the pilot was to improve survey reliability, and responses gained during the pilot suggested that the questions were reliably interpreted in similar ways. Feedback identified opportunities to improve question clarity and user interface. These adjustments were made before data collection began in August 2017. The full survey is included as Appendix 1.

Respondents answered general questions about the hospice before specific questions regarding services they considered to offer social support. Respondents identified services offering social support to people living in the community with life-limiting illness. The survey allowed up to 8 different services to be identified in free text boxes, it then asked further questions about each of these services. Respondents were asked to identify which service they felt offered *most* opportunity for social support and gave detail on the aims, group size, and criteria for attendance of that service in particular.

### Inclusion & ‘exclusion criteria’

Target respondents for this survey were hospice staff involved in the management of outpatient or day care services for adult patients. Hospices offering *only* inpatient or home care were not included. There were two hundred and eight (208) eligible hospices in the UK & ROI and these were invited to participate in the survey. See Appendix 2 for eligibility breakdown.

### Data collection

Data collection began in August 2017 and ended in May 2018. The survey was promoted via eHospice (an email news resource in palliative care) and the mailing lists of the Association of Palliative Day Services, and the All Ireland Institute of Hospice & Palliative Care. Email and telephone contact was made with each hospice within the first six months, with reminder emails sent to non-respondents on up to two occasions.

A contact from each hospice was emailed an invitation to participate, with a link to the survey. If contact details were available from the hospice’s website, this was the lead/manager of hospice day services, or the equivalent identifiable role; or the director of the hospice itself. If specific contact details were not available, a generic email address was used, and the email marked for the attention of management staff of day services, day care, or outpatient services. Following this, telephone contact was made with each site, to identify the most relevant member of staff, and introduce the survey directly to them if possible.

Potential survey respondents were provided with information about the research project and the researcher’s contact details for further queries. All survey respondents consented to participate in the study. No financial or other incentive was provided.

### Analysis

Survey responses were exported from SurveyMonkey at the end of data collection. Partial responses were included in analysis, but missing item data was not imputed or pursued. There were a small number of instances in which two responses were received from the same hospice – these were reviewed and only the most complete set of responses used. Representativeness of the sample was considered in terms of location, funding, and diagnosis mix.

Questions are summarised independently to each other. Descriptive statistics were calculated in Microsoft Excel. Non-numerical data was grouped thematically using both manual coding and NVivo12. Respondents identified services provided by their hospice and these answers were grouped like-for-like in a progressive manner to summarise this information in stages to reach an overview of the different services offered to facilitate social support (as identified by these survey respondents and interpreted by these researchers). This process is depicted in the accompanying figure. Supplementary results tables are provided.

## Results

### Respondents

**Fig. 1:**
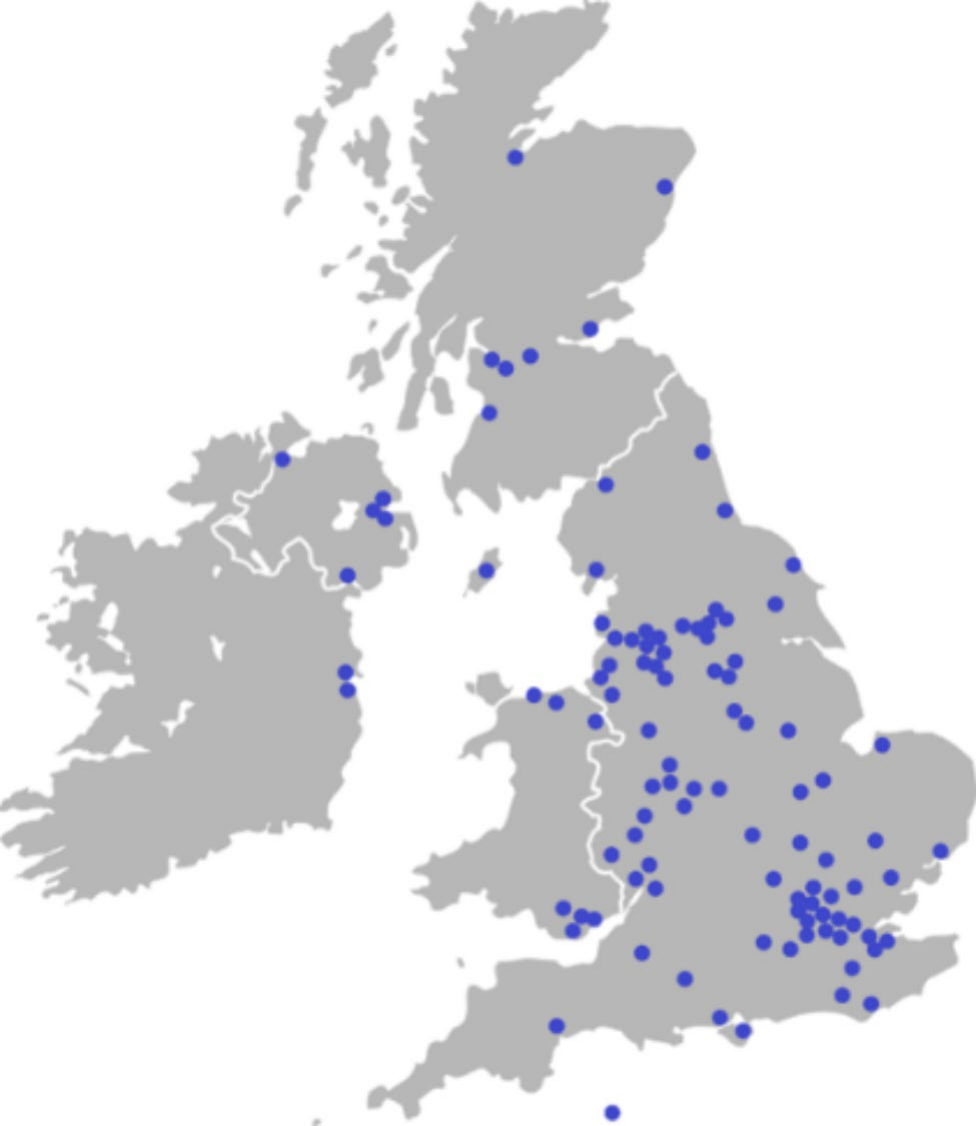
Survey respondent location as shown on a map of the UK & ROI

Management staff at 208 hospices were invited to participate in the survey. Responses were received from 103 hospices (response rate 49.5% - see Appendix 2). These were from England (82), Scotland (7), Wales (7), Northern Ireland (5) and the Republic of Ireland (2) (Fig. 1; Table 1). Most respondents (83.5%) were in management or senior leadership positions. Nursing was the most common discipline represented, followed by allied health professionals, clinicians, and social workers. Unless otherwise stated, n = 103.

**Table 1:** Survey response rate by country

Country	Response rate
England	57.3%
Scotland	46.7%
Wales	53.9%
Northern Ireland	100%
Republic of Ireland	28.6%

### Hospice funding and range of diagnoses

Most hospices in this sample had a mixture of statutory and fundraising income, receiving some funding from the NHS or local authorities (mean average = 31.3%, range 0-100%). Four hospices received no statutory funding, four were fully funded by the NHS or local authorities.

**Fig. 2:**
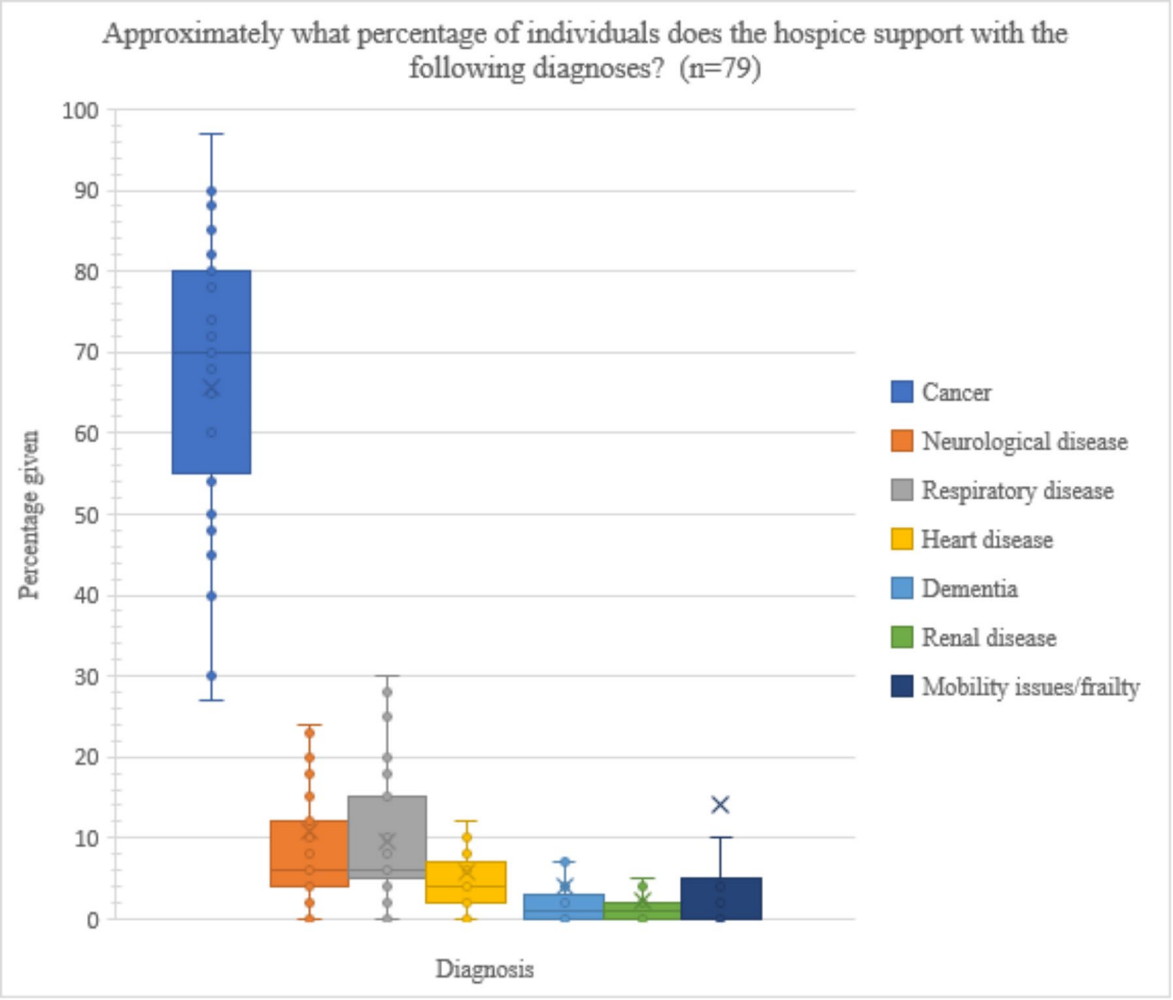
Box plot presenting diagnosis mix as reported by respondents

In an optional question, respondents (n = 79/103) estimated the proportion of diagnoses that the hospice supports overall (Fig. 2, Table S1). Cancer was by far the most common (mean average = 65.5%), followed by mobility issues/frailty (14.2%), neurological disease (10.7%), and respiratory disease (9.6%). There was high variation in the extent to which cancer dominated the diagnosis mix, but the most extensive variation between respondents was in the category of mobility issues and frailty.

### Service locations, transport, and payment

Respondents were asked if any hospice services were offered in locations outside the hospice building (Table S2). Most frequently this was in people’s homes (74.2%); but also in community centres, libraries, and other non-religious buildings (23.7%); outdoors in gardens or parks (16.5%); and in religious buildings such as church halls (13.4%).

At the time of data collection, twenty-seven of the hospices in this sample (26.2%) were linked to a compassionate community project, fifty-two were not (50.5%), and twenty-four respondents were unsure (23.3%).

Transport to and from the hospice was provided by almost all hospices in the sample (96.1%) (Table S3). This was usually provided by volunteers (77.7%) or paid staff (13.5%). Five hospices offered transport provided by a different organisation (4.8%), and four did not offer transport (3.9%).

Most hospices in this sample (75.7%) did not ask for payment towards any services (Table S4). If payment was requested, it was most commonly an optional donation per session (11.7%) or a set charge for lunch/catering (11.7%). Occasionally, there was an optional donation towards lunch (4.9%), an optional donation towards transport (3.9%), or a set charge for some activities/services (3.9%).

### Staff and volunteer roles

The survey asked what roles were present and whether they were carried out by paid staff, volunteers, or both (Table 2). Nurses, administrators, and catering staff were the most common paid roles. Frequent volunteer roles were complementary therapy, befriending and hairdressing. Doctors, pharmacists, and social workers were the least likely to be voluntary. When counting both paid staff and volunteers, over 80% of responses indicated the presence of: nurses, administrators, cleaners, complementary therapists, catering staff, religious leaders, doctors, psychotherapists, and physiotherapists.

**Table 2:** What roles are carried out at the hospice?

Role	Total % with this role	% w/ staff in role	% w/ vols in role	% without role
Nurse	93.2	93.2	3.9	6.8
Administrator	93.2	92.2	54.4	6.8
Cleaner	93.2	87.4	7.8	6.8
Complementary therapist	90.3	75.7	61.2	9.7
Catering staff	89.3	89.3	34.0	10.7
Chaplain/other religious leader	88.3	69.9	48.5	11.7
Doctor	85.4	85.4	1.0	14.6
Psychotherapist	79.6	76.7	34.0	20.4
Physiotherapist	78.6	78.6	6.8	21.4
Social worker	69.9	69.9	1.0	30.1
Occupational therapist	67.0	65.0	2.9	33.0
Hairdresser	65.0	19.4	53.4	35.0
Creative activity leader	64.1	50.5	26.2	35.9
Befriending	62.1	13.6	61.2	37.9
Pharmacist	58.3	58.3	0.0	41.7
Mindfulness	53.4	36.9	28.2	46.6
Other activity leader	41.7	29.1	23.3	58.3

### What services offer social support?

**Fig. 3:**
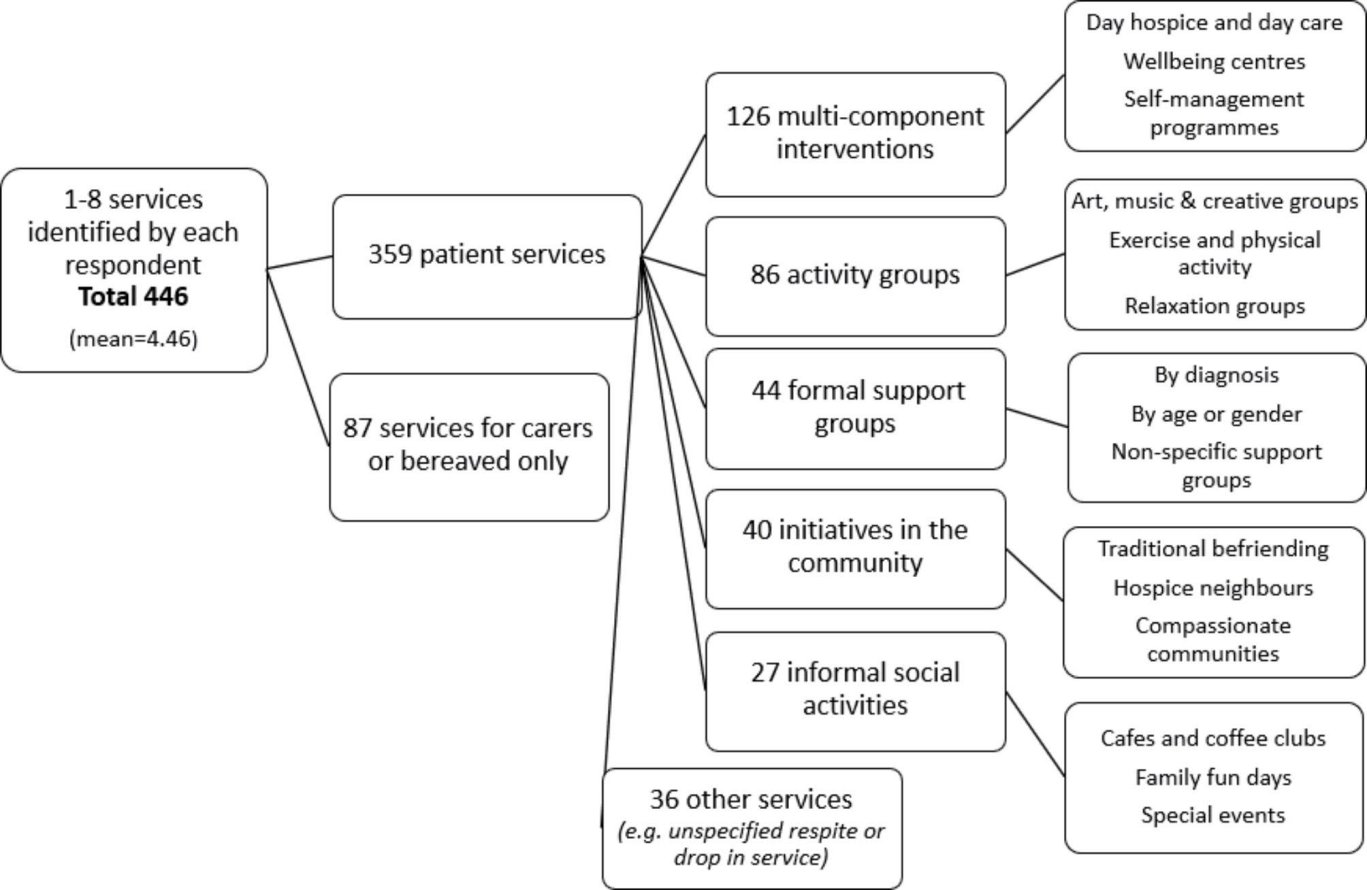
All categories of hospice services that offer social support, as identified by survey respondents answering the question: “How does the hospice offer social support?”

One hundred respondents (n = 100) identified 446 hospice services in total that they considered to offer social support to adults with life-limiting illness. Some services were for carers or bereaved only (19.5%) and were excluded. The remaining three hundred and fifty-nine services for patients were grouped into like-for-like themes (Table S5). This included some befriending and compassionate community projects oriented around the home (9%). The services for patients outside of their home were categorised into four broad headings: multicomponent interventions, activity groups, formal support groups, and informal social activities. The detail of the categories and the services identified within them is presented in Fig. 3.

Multi-component interventions, including palliative day care and self-management programmes, were the most common. Activity groups were the most wide-ranging. Commonly these involved arts, crafts, music or singing. Different forms of exercise, including dance and yoga, were offered in hospices across the country. Groupwork was associated with complementary therapy practice as well as with relaxation and mindfulness. Also reported were activity groups on cooking, computer-mediated communication, and gardening or horticulture, but these were less common. Formal support groups were often organised by diagnosis (45.4% of those identified), but also by age or gender (15.9%), and there were many non-specific support groups (40.9%).

In total, 175 (39.2%) of all identified services operated as ‘drop in’. Social activities (including cafes and coffee clubs, social programmes, friendship groups, family fun days and special events or excursions) were very commonly ‘drop in’ - in 70.4% of cases. A notable proportion of activity groups and support groups (43% and 40.9% respectively) operated as ‘drop in’. Multicomponent interventions were offered on a drop-in basis in 20.6% of the services identified by this survey. A significant proportion of respondents (30%) did not identify any services to operate on a ‘drop in’ basis.

In total, 54 (11.7%) of services were organised by volunteers, carers or patients. Eleven of these were for carers or bereaved only. Volunteers, carers, or patients were most likely to organise social activities (29.6% of those identified) and this figure was lower for other categories, but not absent – 15.9% of support groups, 7% of activity groups, and just 4% of multicomponent interventions. Cost data was rare, respondents indicated that they had this information available for just 7% of all identified services.

### Which is the ‘most social’?

Respondents were asked to choose which of the services identified offered palliative care patients the most opportunity for social support, in their opinion (n = 88). The most frequently selected category was that of multicomponent interventions, chosen by 62.5% of the respondents overall and 43.7% of those which identified any services in this category (Table S6). Social activities, if present, were more likely to be selected as ‘most social’ – chosen by 14 respondents which is 51.9% of those hospices which identified any activities in this category. It may be notable that this percentage is not higher - some participants said that (often clinical) multicomponent interventions were ‘most social’, even when their hospice provided other less medical services .

A question on group size suggests between ten and twenty people typically attend these services per session. A small number of respondents (4.3%) indicated that the ‘most social’ service at their hospice is typically attended by over forty people, but maximum group size is usually between ten and twenty people. Capacity is determined by the physical space available, the dependency of the expected patients, and the activity involved. Some respondents over-book the service because it is so expected to have non-attenders; some under-book to allow for increased patient complexity on the day.

The ‘most social’ services were usually provided during weekdays, however 12% of hospices had their ‘most social’ service at the weekend. The services that operated seven days a week (4.3%) were befriending, compassionate communities, and a social programme.

When asked how people find out about the service, many respondents described formal referral pathways (49.5%) (Table S7). Patients most commonly hear about services through health or social care professionals, who can make a formal referral into the hospice. However, self-referral or drop-in models of access were not uncommon. Respondents reported a range of promotional strategies including advertising through local newspapers, GP surgeries, charity shops, tea rooms and supermarkets. The hospice website, email newsletters and the role of social media were also frequently mentioned.

More than half of the ‘most social’ services reported no outcome measurement. 15% reported the introduction of all or part of the OACC suite of measures. One hospice used the DeJong Gierveld loneliness scale at a drop-in coffee club, but on follow-up they reported it had been difficult to maintain in practice in that informal setting.

Respondents were asked for the stated or official aim for their ‘most social’ service in a free text box (n = 92). Often these aims were multi-faceted, reflecting the multicomponent nature of many of these services (Table S8). Thirty-seven respondents (40%) referred explicitly in their aims to social support, or an unambiguously social objective (including the facilitation of peer support and the opportunity to meet others in a similar situation; in a supportive, safe, and welcoming environment; reduction of social isolation; time out of the house for enjoyment or relaxation; and emotional support). However, it was not unusual for the stated aims to omit social support, despite being considered the ‘most social’ by the respondent. This was particularly the case for multicomponent interventions facilitating clinical or nursing input, specialist palliative care, or symptom self-management. Other aims identified for the ‘most social’ service were psychotherapy and spiritual support; holistic or individualised care; advanced care planning; patient rehabilitation; promoting independence and managing at home; carer respite; and community building.

## Discussion

The survey aimed to understand what services facilitated social support for adults living in the community with life-limiting illness. Previous literature describes palliative day care as relieving isolation by providing an opportunity to get out of one’s home and as such this survey focussed on hospice day and outpatient services occurring outside of the patient’s home. The social significance and outcomes of at-home services, or those occurring within inpatient or residential settings, or those occurring within community or public spaces, are all worthy of separate study.

### Summary of findings

The present survey reports a diversity of approaches used by hospice day services to facilitate social support for patients outside of their own homes. Many respondents identified numerous different services at their hospice to be offering social support, including those with a medical focus. The sample of hospices were from across the UK & ROI and were broadly representative in terms of funding and diagnosis mix with the hospice sector as a whole.

On average, respondents identified between 4 and 5 services each. Most (but not all) of these services were located within the hospice main building, and the hospice offered transport and catering, usually for free, although roughly 25% requested a donation for some component or charged for lunch. Drop-in models were not unusual. Promotion activities sometimes used diverse media formats and inter-organisational working. Self-referral was widespread, but the majority still required formal referral from a healthcare professional to access the services. Multidisciplinary teamworking described by the present survey indicates a mix of clinical and supportive input, with over 80% having nurses, administrators, cleaners, complementary therapists, catering staff, religious leaders, doctors, psychotherapists, and physiotherapists.

The most commonly indicated services were multi-component interventions, and patient activity groups (frequently exercise, music, art, or relaxation). This was followed by formal support groups and lastly informal social activities such as coffee clubs. Social aims included opportunities to meet others in a similar situation and gain peer support in a safe, welcoming, and supportive environment – time out of the house for relaxing or socialising was also prominent. This aligns with previous conclusions that getting out of the house was necessary for alleviation of both physical and psychosocial isolation in palliative care patients.[48] Most often, however, the stated aims of ‘most social’ services did not reflect social aspects - instead emphasising clinical input, symptom management, and access to specialist components. Survey respondents considered the social support obtained within clinical services to be important, even when those services did not formally have a social aim. This suggests social support could be an important aspect of many group settings in hospice day services.

Outcome measures tended not to be collected in the ‘most social’ services identified by this survey. Some of these are open-door and variable in attendees – truly ‘drop in’ – which might offer advantageous flexibility for some patients and their families. In these services, collecting patient outcomes could represent an additional set of paperwork in a relatively nonclinical and informal setting. Cost data was even rarer, which poses challenges for the demonstration of patient outcomes and comparison of different service models. Patient reported outcome measures are increasingly used in traditional palliative day care, but the acceptability and feasibility of outcome measurement is less established in newer settings.[61]

### Comparison with previous surveys

An early telephone survey of palliative day care services (1998) found that all responding day care centres provided social and psychological support for patients, together with a range of other services.[32] At the time, almost all palliative day care services treated mostly cancer patients. In the present survey, cancer remains dominant, but less so: mean average across sites was 65.6% and variation was high (3–97%), with only four respondents reporting their diagnosis mix to be 90%+ cancer.

A regional survey (2000) reported hospice day services to have social, psychological, physical, and (to a lesser extent) existential objectives.[26] 90% of patients had cancer, and on average a quarter of patients attended for over a year. Social interaction was a reason for referral in all centres, and companionship and support were the most commonly reported benefits to patients. Rehabilitation was not considered an explicit aim of palliative day care at the time, unlike today. 50% of the palliative day care centres in the Thames region offered services specifically for younger people – but this part of the UK included is more densely population and better provided with palliative care services than many regions.[26] The present survey, covering a broader geography, found fewer services for young people.

The present survey reports a similar profile of personnel roles to these previous surveys, in that most centres had doctors, nurses, chaplains, managers, aromatherapists, and hairdressers. Results here indicate occupational therapists, social workers, and psychotherapists are becoming more common. A contemporary survey (2019) reports in agreement that most hospices have access to specialist psychotherapists, but notes many respondents felt psychological care offered was not wholly adequate.[56] In the current sample, the most variation was found in whether mindfulness practitioners and in-house pharmacists were present.

An anonymous online survey (2013) found that 60% of respondents (66% response rate) were active in public health approaches to palliative care, specifically relating to community engagement, awareness raising, compassionate communities, or ‘Dying Matters’ events.[52] Working with schools, local businesses, faith organisations, and local events might increase the reach of hospice messaging, but approaches to working in partnership with local communities are under-researched. The present survey reports that a quarter of respondents offered services in community centres, libraries, or other religious and non-religious buildings. Outdoor settings such as community gardens or public parks were reported by 16.5% of respondents. Six of the responding hospices had multiple locations, including satellite sites, and two were working in schools or universities in a way that included patients.

An online survey of volunteer activity in UK adult hospices and specialist palliative care services (2014) highlighted the social nature of the palliative care volunteer role.[31] Our survey agrees that volunteers are involved in social roles, such as befriending, in more than half of the hospices surveyed (61.2%), and providing transport for most (77.7%). However, the present survey also shows volunteers to be giving professional or therapeutic expertise: volunteers were complementary therapists, administrators, hairdressers, chaplains or other religious leaders, psychological therapists, mindfulness or meditation practitioners, and leaders of creative or other activity groups. These roles can involve a high degree of interpersonal interaction, but also have additional skillsets and management or supervision requirements - which might relate to the high proportion of respondents with both paid staff and volunteers in a particular role.

Challenges to palliative day care arise from a narrow public perception of palliative care, insufficient occupancy and/or unclear referrals, and insecure funding models.[63] We report very similar challenges, and add negotiating transport and distance – hospices providing transport to attend do not have resources to do this for all services or for all patients and may struggle to provide reliable transport when dependent on volunteers. A recent study of costs in three palliative day care services in the UK (2020) found that the contributions of volunteers (as complementary therapists, drivers, catering staff or hairdressing) was equivalent to roughly a third (28-38%) of running costs per day per patient.[58] The hospice sector depends upon volunteers, and thus community engagement that increases the visibility of hospice volunteering could be considered essential to workforce planning.

Multidisciplinary teams benefit from being able to offer ‘multimodal’ therapeutic options, team-working, and multiple perspectives on a patient - however effective communication and mutual respect within the team is essential.[59] The composition of a ‘traditional’ palliative care team is not clear in this survey, or generally - the range of professional and voluntary members continues to evolve over time, with different hospices also having different interpretations of teamwork models and role overlap. Multicomponent interventions such as palliative day care can welcome people with complex clinical and emotional needs, but this requires multiple strands of expertise and a team of professional perspectives working together. This can have a high cost per patient that may become increasingly untenable if issues of access and financial sustainability are unresolved. However, fluctuations in the statutory funding available and expectations attached are clear obstacles to long-term planning.

### Strengths and limitations

This online survey received responses from 103 hospices in the UK and ROI (response rate estimated 49.5%, calculated from the number of unique responses divide by the number of eligible hospices). Recommendations for a minimum acceptable survey response rate vary from 50–75%.[64] It was not expected that every hospice we contacted would have an interest in social support. The response rate achieved here is roughly comparable with other national surveys of the sector.[52].

Representativeness of the sample to the sector as a whole was considered according to: location by country, level of statutory funding, proportion of cancer to non-cancer diagnoses served. The sample was representative to the sector as a whole in terms of average statutory funding and the proportion of non-cancer patients. However, fewer responses were received from the Republic of Ireland (2 out of 7 eligible hospices) than from the other countries included.

Although the sample is representative in some domains, generalisability is limited by non-response bias - respondents are more likely than non-respondents to have an interest in social support. While some hospices are research-active and running their own research projects ‘in house’,[65] it is not established what proportion of hospices are open to research and, in this case, would encourage or discourage participation in online research surveys.

All surveys are vulnerable to respondent issues in that we assume respondents are reliable, and that the answers they provide are valid. We depend on each respondent’s understanding of whether social support is facilitated within a certain setting, rather than objective empirical observation (i.e., the data is self-reported and not validated). We did not ask all questions about all services, and we cannot conclude that we captured every relevant service.

The findings of this survey contribute comprehensive detail of a vibrant area of hospice practice. Results demonstrate hospice day services to reflect multiple aims, multi-professional teams, volunteer roles, and differences in transport and catering provision. Free text answers were useful to indicate the extent of change within the sector. Overall, the survey was successful in its aim to generate an overview of hospice day service models that might facilitate social support for patients. The picture painted is one of diversifying hospice day services, with clear interest in social support but without consensus on aims. This is a top-level description and service provision is deeply affected by the Covid-19 pandemic and its ongoing impact on the health and care workforce. A deeper approach is required to gain an understanding of how the significance of social support to patients relates to the potential outcomes of holistic palliative care.

## Conclusion

The current survey aligns with previous reports and the holistic philosophy of hospice care that physical, emotional *and* social support is often provided.[26, 32] The responding hospices all offered social support to patients, in-person, and often this was alongside nursing, spiritual, clinical, psychological and/or volunteer input. The range of activities may be underpinned by differences in hospice ethos - philosophies that shape the setting, and thus the type of social support gained - such that it can be hard to gain insight into some of the social sides of a service without sitting in it, for example, with direct researcher observation.[30].

Professional and volunteer roles can reflect different goals of the intervention (e.g., clinical or symptom-focused treatment, community engagement, self-management education, or functional rehabilitation). What these services have in common is some kind of social interaction with other people - including but not necessarily limited to other patients - and thus the opportunity to gain social support. Since people ‘vote with their feet’, in that unsuccessful groups get smaller,[62] social experience will always have some influence on the acceptability and therefore effectiveness of group interventions. At the very least, social objectives should not be subordinate to healthcare objectives within the social settings of palliative care [30, 50]. Yet where the objectives of (funding) health and social care agencies lead to the collection of healthcare outcomes, the success of groupwork is not measured in therapeutic or social gain.

This survey captures a moment in time in which the hospice sector was diversifying its provision and engaging in critical reflection of its social offering. Variability between hospice interventions and the range of possible consequences for patients which can make the strengths and limitations of different service models difficult to compare.[63] Many respondents reported that they had recently redesigned or are about to redesign their model of services, considering social support to be an important and perhaps under-recognised part of the work they do. Others were in the process of reviewing their day services, expanding what they offer to meet different levels of patient need and provide choice.

Alongside (and possibly propelling) the diversification of hospice day services was a shifting financial landscape, with future funding and staffing levels in uncertainty. This uncertainty has only multiplied in the time since data collection. Many hospice day services have found new approaches to facilitate social connection at a distance, including activity packs by post and online group meetings. But the significance of social support within an in-person setting remains poorly understood. More knowledge is needed to understand effectiveness of social support interventions in palliative care - as the building blocks for the next evolution of the hospice movement.

## Electronic supplementary material

Below is the link to the electronic supplementary material.

Supplementary Material 1

Supplementary Material 2

Supplementary Material 3

## Data Availability

Full datasets generated during this study are not publicly available due to the identifiable details present therein. Research materials and summarised data to support the findings are included in supplementary files. Further queries can be directed to the corresponding author.
