# Supplementary material for: A survey of hospice day services in the United Kingdom & Republic of Ireland : how did hospices offer social support to palliative care patients, pre-pandemic?
Source: BMC Palliat Care. 2022 Oct 5;21:170. doi: 10.1186/s12904-022-01061-9 (PMC9532229; doi:10.1186/s12904-022-01061-9)
Supplement: Supplementary file 1 — Supplementary Material 1 [file 12904_2022_1061_MOESM1_ESM.docx]

## Appendix 1: Survey

Contact details

*1. Your name:

*2. Job title:

*3. Contact telephone number:

*4. Contact email address:

*5. Please indicate here if you are happy to be contacted to take part in the next stage of the research. You can withdraw from the research at any time.

Yes

No

About the hospice

*6. Hospice name:

*7. Hospice postcode:

8. **Approximately** what percentage of individuals does the hospice support that have the following primary diagnoses?
(optional question)

Cancer

Respiratory disease e.g. COPD

Neurological disease e.g. MND, MS.

Heart disease

Renal disease

Mobility issues/frailty

Dementia

HIV/AIDS

Other

*9. What percentage of funding is currently provided by statutory sources (e.g. the NHS or local authorities)?

| **0** | **50** | **100** |
| --- | --- | --- |

*10. Does the hospice offer **transport**for people attending its services?

No

Yes (provided by volunteers)

Yes (provided by paid staff)

Yes (provided by a different organisation)

*11. Does the hospice offer services to people who **do not** have a life-limiting illness ?

Yes

No

Not sure

*12. Is the hospice linked with any **compassionate communities** projects?

Yes

No

Not sure

*13. Does the hospice offer any services that take place **outside of the hospice building**? i.e. in a different location such as a library or community garden. Please tick all relevant options.

No

Community centre, library or other non-religious building

Religious building such as a church hall

Outdoors - e.g. a community garden or park

In people's homes

Other (please specify – free text box)

*14. Does the hospice ask for**payment** towards any of its services? Please tick all relevant options.

No

Set charge per session

Optional donation per session

Charge for transport

Charge for lunch/catering

Charge for some activities

 Other (please specify – free text box)

What roles are carried out at the hospice?

*15. Please indicate whether the roles below are provided by **paid staff, volunteers**or **both**. If a service is not available at the hospice, please select 'not applicable'.

|  | **Paid staff** | **Volunteers** | **Both paid staff and volunteers** | **Not applicable** |
| --- | --- | --- | --- | --- |
| Doctor |  |  |  |  |
| Nurse |  |  |  |  |
| Physiotherapist |  |  |  |  |
| Occupational therapist |  |  |  |  |
| Psychotherapist, counsellor or other mental health support |  |  |  |  |
| Pharmacist |  |  |  |  |
| Social worker |  |  |  |  |
| Chaplain/spiritual worker |  |  |  |  |
| Hairdresser or beautician |  |  |  |  |
| Creative activity leader |  |  |  |  |
| Other activity leader |  |  |  |  |
| Complementary therapist |  |  |  |  |
| Mindfulness or meditation practitioner |  |  |  |  |
| Catering |  |  |  |  |
| Cleaning |  |  |  |  |
| Administration tasks |  |  |  |  |
| Befriending |  |  |  |  |

How does the hospice offer social support?

We are looking for information on any services that are currently managed by the hospice that offer people living in the community the opportunity to spend time with other people. These can be any hospice or community-based services - for example day care, respite, community centres, empowerment centres, support groups or group therapy, creative/craft groups, diversionary activities or community engagement activities.

Please state any services that you think may be relevant to our research. You may state up to eight different services, but please enter at least one.

*16. Service 1

17. Service 2

18. Service 3

19. Service 4

20. Service 5

21. Service 6

22. Service 7

23. Service 8

24. Do any of these services operate as a **'drop in'**? Please tick all relevant boxes, or 'none'.

{{ Q16 }}

{{ Q17 }}

{{ Q18 }}

{{ Q19 }}

{{ Q20 }}

{{ Q21 }}

{{ Q22 }}

{{ Q23 }}

**None**

25. Are any of these services **organised** by volunteers, carers or patients? Please tick all relevant boxes, or 'none'.

{{ Q16 }}

{{ Q17 }}

{{ Q18 }}

{{ Q19 }}

{{ Q20 }}

{{ Q21 }}

{{ Q22 }}

{{ Q23 }}

**None**

*26. In your opinion, which of these services offers the **most** opportunity for social support? You will be asked some more questions about this service.

{{ Q16 }}

{{ Q17 }}

Note: conditional formatting was used during survey design. Here, {{ question number }} is replaced by respondent’s answers to Q16-23 & Q26

{{ Q18 }}

{{ Q19 }}

{{ Q20 }}

{{ Q21 }}

{{ Q22 }}

{{ Q23 }}

*27. What is the hospice’s stated **aim** in offering this {{ Q26 }} service?

(free text box)

*28. **When** does this service take place? (tick boxes)

|  | Morning | Afternoon | Evening |
| --- | --- | --- | --- |
| Monday |  |  |  |
| Tuesday |  |  |  |
| Wednesday |  |  |  |
| Thursday |  |  |  |
| Friday |  |  |  |
| Saturday |  |  |  |
| Sunday |  |  |  |

*29. What is the **typical number of people** that attend this service per session?

0-10

11-20

21-30

31-40

41-50

50+

*30. What is the **maximum number of people** that can attend this service per session?

(free text box)

31. How do people find out about this service? If they are formally referred, please state how.

(free text box)

*32. Please give detail of any **exclusion criteria**. If none, please state 'none'.

(free text box)

*33. Is there a **discharge policy** or a limit on how long people can attend?

Yes

No

34. Please give detail on discharge policy

(if applicable)

Maximum number of sessions:

Maximum time frame:

Other discharge policy:

*35. What (if any) **outcomes** are measured? If possible, please indicate how frequently and by who. If none please state none.

(free text box)

36. Do you have an **estimate of cost** to provide any of these services? (eg per patient, per session or annually). If this information is not available, please select No.

{{ Q16 }}

{{ Q17 }}

{{ Q18 }}

{{ Q19 }}

{{ Q20 }}

{{ Q21 }}

{{ Q22 }}

{{ Q23 }}

**No**

37. If yes please state below

Final questions

38. Do you have anything else you would like to tell us about services offering social support at your hospice?

(free text box)

39. Do you have anything else you would like to tell us about the hospice or its services more generally?

(free text box)

40. In the space below, you may provide an URL for any promotional material or further information

(free text box)

41. In the space below, you may upload any promotional material or further information

(file upload)
