# Supplementary material for: A survey of hospice day services in the United Kingdom & Republic of Ireland : how did hospices offer social support to palliative care patients, pre-pandemic?
Source: BMC Palliat Care. 2022 Oct 5;21:170. doi: 10.1186/s12904-022-01061-9 (PMC9532229; doi:10.1186/s12904-022-01061-9)
Supplement: Supplementary file 2 — Supplementary Material 2 [file 12904_2022_1061_MOESM2_ESM.docx]

## Appendix 2: Identifying the Sample

There were two hundred and eight (208) eligible hospices in the UK & ROI and these were invited to participate in the survey. The Hospice UK directory was considered the most comprehensive list of hospices in the United Kingdom and so was used as a framework to identify respondents and calculate response rate. The Hospice UK directory (accessed 11/05/2017) listed 288 hospice addresses in the UK; of which 51 are children’s hospices only, 12 inpatient only, and 10 homecare only. Some hospices operate from more than one location (commonly two, but up to eight including satellite sites) – 14 entries in the directory were for this purpose duplicates, where more than one address was listed for the same organisation. There are nine hospice organisations in the Republic of Ireland providing support for adults with life-limiting illness, but two of these provide only homecare or inpatient care and so were not eligible for this survey. Therefore, the total number of hospices eligible to participate in this survey was calculated as 208:

(288 total in UK +9 total in ROI =297 total) MINUS (51 children’s + 21 homecare/inpatient +14 duplicates =89) EQUALS 208

Note that Hospice UK have figures from 2011.[[40](#_ENREF_40)] These are calculated differently because their directory includes hospital palliative care (NHS) and Macmillan (national charity), and also counts multiple locations by the same provider individually. Their directory suggests 275 palliative day care locations in total. Estimates for this survey have used hospice websites and conversations with management staff during survey dissemination, as sources of up-to-date information on service provision (i.e. at the time of data collection, 2017/18).
