# Supplementary material for: A survey of hospice day services in the United Kingdom & Republic of Ireland : how did hospices offer social support to palliative care patients, pre-pandemic?
Source: BMC Palliat Care. 2022 Oct 5;21:170. doi: 10.1186/s12904-022-01061-9 (PMC9532229; doi:10.1186/s12904-022-01061-9)
Supplement: Supplementary file 3 — Supplementary Material 3 [file 12904_2022_1061_MOESM3_ESM.docx]

Supplementary Tables

Table S1: Diagnosis mix of individuals supported by the hospice (n=79)

| **Diagnosis** | **Mean** | **(Range)** |
| --- | --- | --- |
| Cancer | 65.6 | (3-97%) |
| Neurological disease (e.g. MS, MND) | 10.7 | (0-80%) |
| Respiratory diseases (e.g. COPD) | 9.6 | (0-40%) |
| Heart disease | 5.9 | (0-40%) |
| Dementia | 3.9 | (0-50%) |
| Renal disease | 2.2 | (0-30%) |
| Mobility issues/frailty | 14.2 | (0-100%) |
| HIV/AIDS | 0.2 | (0-2%) |

Table S2: Locations of services offered outside of the hospice building

| **Response** | **Frequency** | **% of total** |
| --- | --- | --- |
| No services offered elsewhere | 21 | 21.6% |
| In people’s homes | 72 | 74.2% |
| Community centre, library, or other non-religious building | 23 | 23.7% |
| Outdoors – e.g., in a community garden or park | 16 | 16.5% |
| Religious building such as a church hall | 13 | 13.4% |
| Healthcare settings (hospitals, care homes, GP practices) | 9 | 8.7% |
| Other hospice locations (satellite sites, separate centres) | 6 | 5.8% |
| Additional community teams | 5 | 4.9% |
| Schools/universities | 2 | 1.9% |
| Other: hospice retail shops, other hospices, hotels | 3 | 2.9% |

Table S3: Percentage of hospices offering transport

| **Response** | **Frequency** | **% of total** |
| --- | --- | --- |
| Yes – (provided by volunteers) | 80 | 77.7% |
| Yes – (provided by paid staff) | 14 | 13.5% |
| Yes – (provided by a different organisation) | 5 | 4.8% |
| No | 4 | 3.9% |

Table S4: Payment requested for hospice services

| **Response** | **Frequency** | **% of total** |
| --- | --- | --- |
| None | 78 | 75.7% |
| Optional donation per session | 12 | 11.7% |
| Charge for lunch/catering | 12 | 11.7% |
| Optional donation for lunch/catering | 5 | 4.9% |
| Optional donation for transport | 4 | 3.9% |
| Charge for some activities/service | 4 | 3.9% |
| Set charge per session | 3 | 2.9% |
| Charge for transport | 2 | 1.9% |

Table S5: Services identified by survey, with breakdown by categories of 'drop in' services and those organised by volunteers, carers, or patients (n=100)

| **Multi-Component Interventions**  ‘Drop in’: 20.6%  Led by non-staff: 4% | Day hospice, day care, day therapy 82  Wellbeing centres 17  Self-management programmes 27 (Condition-specific eg Fatigue and Breathlessness; + more generic eg Living Well |
| --- | --- |
| **Activity Groups**  Total: 86  ‘Drop in’: 43%  Led by non-staff: 7% | Art and/or craft groups 27  Exercise and rehabilitation 13  Music, singing, choir 11  Relaxation, meditation, mindfulness 9  Tai Chi/Qi Gung/Yoga 6  Complementary therapy groups 5  Gardening/horticulture 3  Knitting 3 Walking 3 Writing 2 Pamper sessions 2  Cooking 1 “Click a friend” 1 |
| **Formal Support Groups**  Total: 44  ‘Drop in’: 40.9%  Led by non-staff: 15.9% | Support groups by diagnosis 20 (Respiratory disease, dementia, MND, heart failure, cancer)  Support or counselling groups (not-specified) 18  Support groups for young adults/teens 2  Support groups for men 3  Support groups for younger women 1 |
| **Social Activities**  Total: 27  ‘Drop in’: 70.4%  Led by non-staff: 29.6% | Cafés and coffee clubs 15  Social programmes/friendship groups 6  Family fun days 3  Special events/excursions inc. young people’s day out 3 |
| **Carers/Bereaved Only**  Total: 87  ‘Drop in’: 60.9%  Led by non-staff: 12.6% | Carers/families 46  Bereaved 41 |
| **Befriending, etc**.  Total: 40  ‘Drop in’: 15% | Befriending/companion service 19  Compassionate communities/neighbours 8  At home hospice services 13 |
| **Other**  Total: 36  ‘Drop in’: 44.4% | Unspecified respite 12 Unspecified drop in 9  Unspecified outreach 2 Transition service 2  Information support inc. advice line 8  User involvement/fundraising group 2  Care homes 1 |

Table S6: Categories of service identified by service respondents as ‘most social’ (n=88)

|  | **Frequency** | **% of responses** | **% of category** |
| --- | --- | --- | --- |
| **Multicomponent** | 55 | 62.5 | 43.7 |
| **Activity group** | 5 | 5.7 | 5.8 |
| **Support group** | 2 | 2.3 | 4.5 |
| **Social activity** | 14 | 15.9 | 51.9 |
| **Befriending etc.** | 7 | 8.0 | 17.5 |
| **Carers/bereaved** | 5 | 5.7 | 5.7 |

Table S7: Referral routes to access ‘most social’ service (n=91)

|  | **Frequency** | **Percentage** |
| --- | --- | --- |
| Formally referred usually by external health or social care professional | 45 | 49.45% |
| Formally referred or can self-refer | 25 | 27.47% |
| Self-referral or drop in model | 12 | 13.19% |
| Internal referral | 9 | 9.89% |

Table S8: stated aims of ‘most social’ service, with frequency of themes (n=92)

| **Social Support**  Peer support / meeting others in similar situation – 9  A supportive environment (inc. safe & welcoming) – 8  Providing social support – 7  Reducing social isolation – 7  Day out/time to relax/for patient enjoyment – 7  Opportunities for social interaction/socialising – 7  Stimulating activities – 4  Emotional support – 3  Opportunity to be heard/for mutual trust – 2 | **Holistic Care**  Specialist palliative care/clinical input – 17  Symptom management 10  Access to psychological therapy or counselling 6  Spiritual support – 4  Advance care planning – 3  Individualised care – 3  Access to complementary therapies – 2  Introduction to hospice – 2  Support throughout ‘the journey’ - 2  Support post-discharge – 1  Signposting – 1 |
| --- | --- |
| **Rehabilitation**  Rehabilitation – 4  Enhancing patient quality of life – 4  Managing at home – 4  Building confidence and independence – 3  Meeting patient goals – 2 | **Other Aims**  Community building – 5  Carer respite – 4  Reduce unnecessary hospital admissions – 1 |
